# GAUSS: a summary-statistics-based R package for accurate estimation of linkage disequilibrium for variants, Gaussian imputation, and TWAS analysis of cosmopolitan cohorts

**DOI:** 10.1093/bioinformatics/btae203

**Published:** 2024-04-17

**Authors:** Donghyung Lee, Silviu-Alin Bacanu

**Affiliations:** Department of Statistics, Miami University, Oxford, OH 45056, United States; Department of Psychiatry, Virginia Commonwealth University, Richmond, VA 23298, United States

## Abstract

**Motivation:**

As the availability of larger and more ethnically diverse reference panels grows, there is an increase in demand for ancestry-informed imputation of genome-wide association studies (GWAS), and other downstream analyses, e.g. fine-mapping. Performing such analyses at the genotype level is computationally challenging and necessitates, at best, a laborious process to access individual-level genotype and phenotype data. Summary-statistics-based tools, not requiring individual-level data, provide an efficient alternative that streamlines computational requirements and promotes open science by simplifying the re-analysis and downstream analysis of existing GWAS summary data. However, existing tools perform only disparate parts of needed analysis, have only command-line interfaces, and are difficult to extend/link by applied researchers.

**Results:**

To address these challenges, we present Genome Analysis Using Summary Statistics (GAUSS)—a comprehensive and user-friendly R package designed to facilitate the re-analysis/downstream analysis of GWAS summary statistics. GAUSS offers an integrated toolkit for a range of functionalities, including (i) estimating ancestry proportion of study cohorts, (ii) calculating ancestry-informed linkage disequilibrium, (iii) imputing summary statistics of unobserved variants, (iv) conducting transcriptome-wide association studies, and (v) correcting for “Winner’s Curse” biases. Notably, GAUSS utilizes an expansive, multi-ethnic reference panel consisting of 32 953 genomes from 29 ethnic groups. This panel enhances the range and accuracy of imputable variants, including the ability to impute summary statistics of rarer variants. As a result, GAUSS elevates the quality and applicability of existing GWAS analyses without requiring access to subject-level genotypic and phenotypic information.

**Availability and implementation:**

The GAUSS R package, complete with its source code, is readily accessible to the public via our GitHub repository at https://github.com/statsleelab/gauss. To further assist users, we provided illustrative use-case scenarios that are conveniently found at https://statsleelab.github.io/gauss/, along with a comprehensive user guide detailed in Supplementary Text S1.

## 1 Introduction

Genome-wide association studies (GWAS) have revolutionized our understanding of the genetic underpinnings of complex human diseases and traits. However, gaining access to individual-level data from these studies is often challenging due to privacy or regulatory constraints. Fortunately, summary statistics from GWAS are widely available and have catalyzed the development of various summary statistics-based genome analysis tools. Popular tools in this category include those for imputing summary statistics for unobserved genetic variants (Lee *et al.* 2013, Pasaniuc *et al.* 2014), prioritizing causal variants (Schaid *et al.* 2018), computing polygenic risk scores (Lewis and Vassos 2020), and conducting transcriptome-wide association studies (Gamazon *et al.* 2015, Lee *et al.* 2015a,b). The effectiveness of these tools often hinges on the accurate estimation of cohort-specific linkage disequilibrium (LD)—the correlation structure among genetic variants. Often, researchers rely on publicly available reference panels like the 1000 Genomes panel, which encompasses genomic data from just 2504 individuals across 26 ethnic groups. This scale and diversity are significantly limited compared to GWASs, which often involve hundreds of thousands of individuals from diverse ethnic backgrounds. Moreover, many of these tools operated solely in Linux command-line environments, posing a steep learning curve that deters many applied researchers from incorporating these valuable resources into their work.

**Figure 1.**
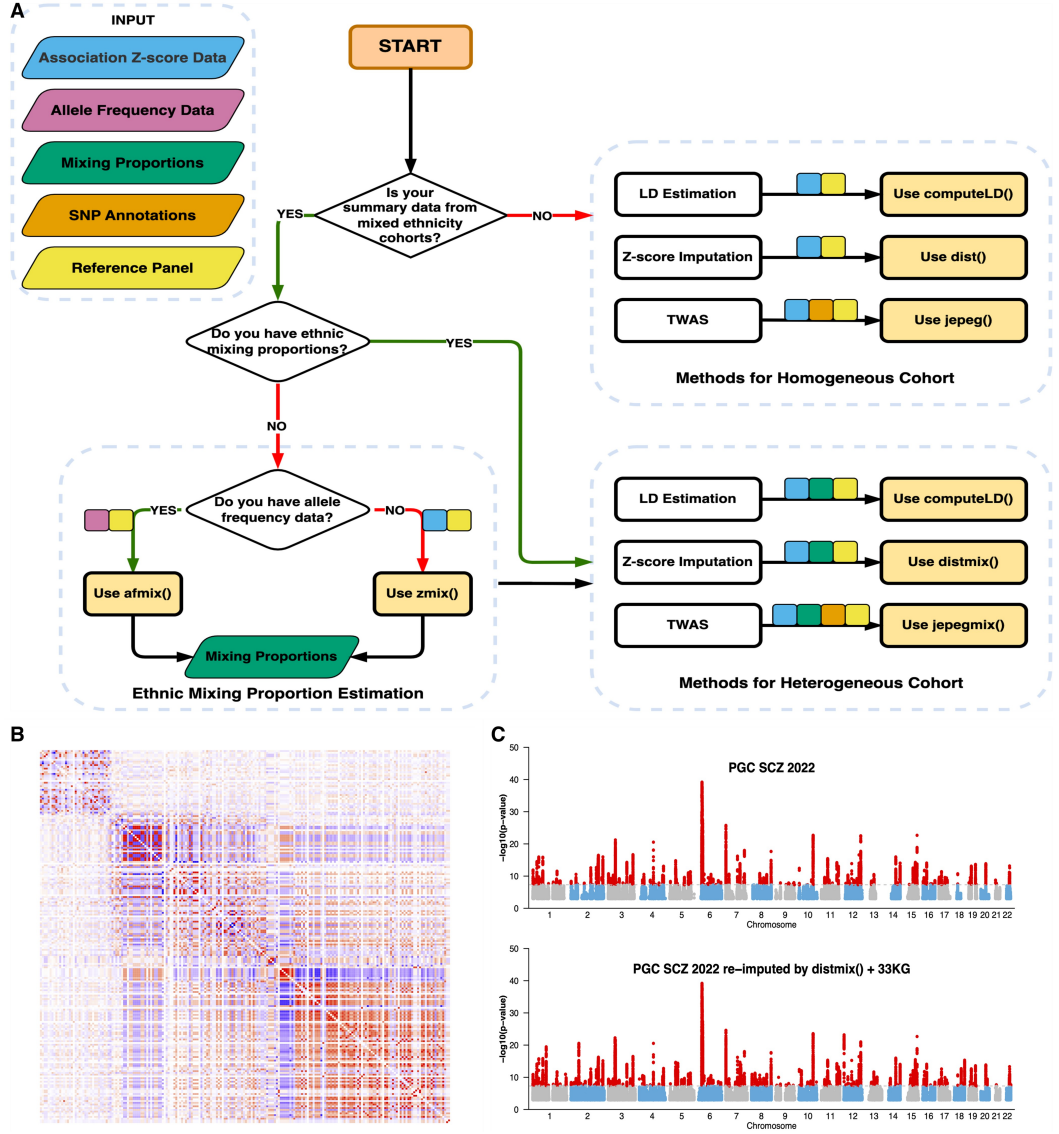
(A) Flowchart depicting GAUSS analytical procedure. GAUSS functions utilize five core input datasets: (i) association *Z*-score data (highlighted in blue), consisting of six columns of variables: SNP ID (rsid), chromosome number (chr), base pair position (bp), reference allele (a1), alternative allele (a2), and *Z*-score (z); (ii) Allele frequency data (colored in purple), encapsulating rsid, chr, bp, a1, a2, and reference allele frequency (af1); (iii) Ethnic mixing proportion data (colored in green), comprising population name abbreviations and corresponding mixing proportions; (iv) SNP annotation data (colored in orange), offering functional annotation information of SNPs; (v) Reference panel data (colored in yellow), offering genotype information for a significant pool of 32 953 individuals from 29 diverse ethnic groups. Small colored squares on arrows pointing to GAUSS functions denote the required input datasets for each function. (B) Ancestry informed LD of the 2022 PGC Schizophrenia GWAS cohorts (chromosome 10: 104–105 Mb). The color intensity represents the strength of linkage disequilibrium (LD) between SNPs. A deeper red indicates a higher positive LD value, whereas a deeper blue denotes a higher negative LD value. (C) Manhattan plots comparing original and re-imputed PGC SCZ 2022 data. The top plot displays the original PGC SCZ 2022 summary statistics, while the bottom plot showcases those re-imputed using the distmix() function, with the Illumina 1M SNP set and the 33KG reference panel. Manhattan plot for Illumina 1M variants from the 2022 PGC SCZ data is provided in Supplementary Fig. S2.

To overcome these limitations, we present **G**enome **A**nalysis **U**sing **S**ummary **S**tatistics (GAUSS), a comprehensive and extendable R package designed for the analysis of multi-ethnic GWAS summary statistics as well as for using its functions to develop new tools. GAUSS (33KG) (Chatzinakos *et al.* 2021), including 20 281 Europeans, 10 800 East Asians, 522 South Asians, 817 Africans, and 533 Native Americans (see Supplementary Text S2 and Supplementary Table S1 for further details). GAUSS equips researchers with a suite of powerful functionalities, including (i) estimating the ethnic composition of a multi-ethnic GWAS cohort (Lee *et al.* 2015a,b, Chatzinakos *et al.* 2021), (ii) computing ancestry-informed linkage disequilibrium (LD), (iii) imputing association *Z*-scores for unmeasured genetic variants (Lee *et al.* 2013, 2015a,b), (iv) performing transcriptome-wide association studies (Lee *et al.* 2015a,b, 2016), and (v) correcting for the “Winner’s Curse” (Bigdeli *et al.* 2016) (see Fig. 1A).

## 2 GAUSS R package

### 2.1 Estimating ancestry proportions in GWAS cohorts

As the diversity of ancestral backgrounds in GWAS increases, accurately estimating the ancestry proportions of the study samples has become more crucial. The estimated ancestry proportions are particularly valuable for summary statistics-based downstream analyses for such multi-ethnic GWAS. For instance, they are essential for computing ancestry-informed LD (Lee *et al.* 2015a,b) and for adjusting allele frequencies for ancestry (Arriaga-MacKenzie *et al.* 2021). Although many classical methods for estimating ancestry composition exist (Pritchard *et al.* 2000, Raj *et al.* 2014), they typically require individual-level genotype data, which is often not publicly available due to privacy concerns. GAUSS addresses this limitation by enabling users to estimate the ancestry proportions of the genetic association studies by utilizing only allele frequencies (AF) through its “afmix()” function (Lee *et al.* 2015a,b). When allele frequencies for the cohort are not available, “zmix()” function can be used. This function is specifically designed to estimate the ancestry composition using only association *Z*-scores (Chatzinakos *et al.* 2021). For more information, see https://statsleelab.github.io/gauss/articles/afmix_example.html

### 2.2 Estimating ancestry-informed LD

Genetic studies often include multi-ethnic samples to enhance detection power and resolution. Accurate estimation of ancestry-informed LD is essential for many tools using summary statistics to analyze such diverse cohorts. To address this need, GAUSS offers the “computeLD()” function, which calculates ancestry-informed LD values using our extensive 33KG reference panel. The algorithm underpinning the LD estimation is elaborated in Section 2.1 of the DISTMIX manuscript (Lee *et al.* 2015a,b). The function generates an LD matrix by calculating a weighted sum of LD values specific to each ethnic group. The LD matrix can be easily integrated into existing summary statistics-based analytical tools that require cohort-specific LD values. The “computeLD()” function requires multiple inputs, such as chromosome number, start and end base pair positions for the window of interest, ancestry proportions, filenames for input and reference panels. Should users not have ancestry proportions, these can be approximated using the “afmix()” and zmix() functions, as detailed in the previous section. For further guidance on the “computeLD()” function, refer to: https://statsleelab.github.io/gauss/articles/computeLD_example.html.

### 2.3 Imputing summary statistics of unmeasured SNPs

As the scale and ethnic diversity of reference panels continue to expand, the need for imputing/re-imputing data becomes increasingly critical. Classical genotype imputation methods (Howie *et al.* 2012, Das *et al.* 2016) are applicable; however, as noted in Section 2.1, they similarly require individual-level genotype data and are computationally very demanding. To address these challenges, GAUSS offer two specialized functions, “dist()” and “distmix(),” for fast summary statistics imputation. These functions are designed for directly imputing summary statistics (i.e. association *Z*-scores) of unmeasured SNPs in both homogeneous and multi-ethnic cohorts using only summary statistics of measured SNPs (Lee *et al.* 2013, Lee *et al.* 2015a,b, Chatzinakos *et al.* 2021). Utilizing the 33KG reference panel, these functions facilitate accurate imputations even for rarer variants(Chatzinakos *et al.* 2021). The “distmix()” function is particularly optimized to handle cohorts with varying ethnic compositions. The imputed summary statistics are readily usable in a wide range of downstream analyses, including transcriptome-wide association studies and meta-analyses. For further details, see https://statsleelab.github.io/gauss/articles/dist_example.html.

### 2.4 Transcriptome-wide association studies

Transcriptome-Wide Association Studies (TWAS) serves as a powerful approach to explore the functional links between genetic variations and complex traits by synergizing GWAS findings with functional annotations. GAUSS facilitates these analyses by incorporating advanced TWAS tools, “jepeg()” and “jepegmix(),” designed for both homogeneous and heterogeneous cohorts, respectively (Lee *et al.* 2015a,b, 2016). While, when compared with other TWAS tools, these two methods also incorporate other functional data, most of information comes from transcriptome data. [We are currently working on integrating in GAUSS the jepegmix2 method (Chatzinakos *et al.* 2018), which solely uses transcriptome data.] The “jepeg()” function performs TWAS by testing for the joint effect on phenotype of expression quantitative trait loci (eQTLs) and functional variants in a gene, leveraging the 33KG reference panel. It requires SNP annotation data that provides essential mapping between SNPs and their corresponding gene-level functional information. It integrates information from the SNP annotation file to construct gene-level test statistics, aiming to identify genes whose expression levels are potentially modulated by genetic variations. The “jepegmix()” function extends the capabilities of “jepeg()” to multi-ethnic cohorts. The results generated by these functions include gene-wise association *P*-values, estimated effect sizes, and other relevant statistics, offering valuable resources for subsequent functional validation or integrative analyses. For more details, see https://statsleelab.github.io/gauss/articles/jepeg_example.html.

### 2.5 Winner’s curse adjustment

In genetic research, ‘sub-threshold’ association signals often have a larger impact on trait variance than statistically significant variants. Accurately estimating these effects is crucial, yet challenging due to “winner's curse” biases. Previously, we've introduced a simple adjustment method called FIQT (FDR Inverse Quantile Transformation), to adjust for these biases using only *Z*-scores, which represent the true means or noncentralities (Bigdeli *et al.* 2016). FIQT is now conveniently integrated as the “fiqt()” function in the GAUSS package. By simply inputting a *Z*-score vector and the minimum nonzero *P*-value output by qnorm() function (10^−320^ by default), the function provides a *Z*-score vector adjusted for the winner’s curse. For more details, refer to https://statsleelab.github.io/gauss/articles/fiqt_example.html.

### 2.6 Applying GAUSS to the 2022 PGC Schizophrenia GWAS

To demonstrate the practical utility of GAUSS, we applied its functions to the 2022 Psychiatric Genomics Consortium (PGC) Schizophrenia (SCZ) GWAS dataset (Trubetskoy *et al.* 2022).

To showcase the applicability of “computeLD()” function, we first computed ancestry-informed LD using the 33KG panel and PGC SCZ 2022 data for a genomic region chr10:104–105 Mb. The genetic correlation between single nucleotide polymorphisms (SNPs) within this region is visually represented via an intuitive heatmap (Fig. 1B). Further evaluating the performance of “computeLD(),” we conducted simulation studies using the SNP list from the 2022 PGC SCZ data and the 33KG panel genotype data. In these simulations, we generated individual-level genotypes for three distinct ancestral composition scenarios. For each scenario, we computed LD using both the simulated genotype data and “computeLD()” function and compared the results (see Supplementary Text S3 for further details). As illustrated in Supplementary Fig. S1, the LD matrices estimated by “computeLD()” function show strong concordance with those obtained from individual-level data.

Next, we utilized GAUSS's distmix() function to re-impute summary statistics for unmeasured SNPs in the dataset. Due to being used by many GWAS studies, we selected Illumina 1M SNPs as our baseline data for measured SNPs (Supplementary Fig. S2). The afmix() function was first applied to estimate the ethnic composition of the study cohort based on this 1M SNP dataset. These estimated proportions, along with the association summary statistics of the measured SNPs, were then used as inputs for the distmix() function to generate association *Z*-scores for SNPs absent in the Illumina 1M array. We run the imputation for 2724 1Mega base pair regions using the Miami University Linux Cluster. The Manhattan plot (Fig. 1C) is created based on the results and compared to the results from the original study. As shown in the figure, distmix() successfully re-imputed the 2022 PGC SCZ GWAS data using the 33KG panel and identified potentially new LD independent association regions that were not found from the original study.

## 3 Conclusion

We have developed an extendable R package GAUSS that allows for (i) re-analysis/downstream analysis of summary statistics from large GWAS and (ii) development of new statistical genetics tools. Built upon robust R and Rcpp frameworks (Eddelbuettel 2013), GAUSS incorporates a comprehensive 33KG reference panel that encompasses genotype data from over 32 953 individuals across 29 distinct ethnic groups. GAUSS is well equipped with functions to (i) estimate ancestral composition, (ii) estimate ancestry-informed LD in specified genomic regions, (iii) impute summary statistics of unmeasured SNPs, (iv) conduct TWAS, and (v) correct for the “Winner’s Curse” biases.

To validate its capabilities, we applied GAUSS to the latest PGC Schizophrenia GWAS, leveraging our expansive 33KG panel to estimate ancestry-informed LD. Moreover, we demonstrated that GAUSS can efficiently update even the most recent GWAS data initially imputed using the more limited 1000 Genomes dataset of 2504 samples. Our 33KG panel refined the dataset and enhanced its resolution significantly.

We believe that, by assisting in accurately analyzing multi-ethnic genetic association studies and providing a strong basis for the development of new tools, GAUSS will help applied researchers in their exploration and understanding of genetic architecture. This could potentially lead to the rapid discovery of many novel risk variants/genes contributing to human disease/phenotype.

## Supplementary Material

btae203_Supplementary_Data
