# Supplementary material for: GAUSS: a summary-statistics-based R package for accurate estimation of linkage disequilibrium for variants, Gaussian imputation, and TWAS analysis of cosmopolitan cohorts
Source: Bioinformatics. 2024 Apr 17;40(4):btae203. doi: 10.1093/bioinformatics/btae203 (PMC11052653; doi:10.1093/bioinformatics/btae203)
Supplement: btae203_Supplementary_Data [file btae203_supplementary_data.docx]

*GAUSS: A summary-statistics-based R package for accurate estimation of linkage disequilibrium for variants, Gaussian imputation and TWAS analysis of cosmopolitan cohorts*

**Supplementary Data**

Donghyung Lee^1,*^ and Silviu-Alin Bacanu

^1^Department of Statistics, Miami University, Oxford, Ohio, USA, ^2^Department of Psychiatry, Virginia Commonwealth University, Richmond, Virginia, USA

*Correspondence: [leed13@miamioh.edu](http://leed13@miamioh.edu)

**Supplementary Text 1.** GAUSS User Guide

**Configuration of Dependencies:** The GAUSS package depends on the GNU Scientific Library (GSL). Before installing GAUSS, users must install GSL. We have provided a detailed guide (<https://statsleelab.github.io/gauss/articles/gsl_installation.html>) for setting up GSL, to assist users in this critical step.

**GUASS Installation:** The GAUSS package can be easily downloaded and installed by following the instructions in `GAUSS Installation` section of the readme file on our GitHub repository (<https://github.com/statsleelab/gauss>) and the Get Started Page (<https://statsleelab.github.io/gauss/index.html>) of our Vignette website (<https://statsleelab.github.io/gauss/>).

**Comprehensive Vignettes for Each Function:** Our GAUSS Vignette Webpage (<https://statsleelab.github.io/gauss/>) hosts extensive vignettes for each function in the GAUSS package, guiding users through various analyses. These include:

1. Estimating Ancestry Proportions (<https://statsleelab.github.io/gauss/articles/afmix_example.html>): A vignette on how to compute ancestry proportions using GWAS summary statistics.
2. Estimating Linkage Disequilibrium (<https://statsleelab.github.io/gauss/articles/computeLD_example.html>): Instructions on using the computeLD() function to compute LD are available here.
3. Imputing Association Summary Statistics (<https://statsleelab.github.io/gauss/articles/dist_example.html>): A guide on using dist() and distmix() functions for imputing association Z-scores of unmeasured SNPs is provided here.
4. Transcriptome-wide Association Study (<https://statsleelab.github.io/gauss/articles/jepeg_example.html>): A detailed walkthrough using jepeg() and jepegmix() functions for this study can be found here.
5. Winner’s Curse Adjustment (<https://statsleelab.github.io/gauss/articles/fiqt_example.html>): Instructions for using the fiqt() function are outlined here.

**Access to Example Datasets:** All example datasets utilized in our vignettes are available in the data folder (<https://github.com/statsleelab/gauss/tree/main/data>) on GitHub.

**Reference Panels:** Comprehensive details and downloadable datasets for both the 1000 Genomes (1KG) panel and the 33000 Genomes (33KG) panel are provided in their respective vignettes. Users can easily access and download these panel datasets from the 1KG panel vignette (<https://statsleelab.github.io/gauss/articles/ref_1KG.html>) and the 33KG panel vignette (<https://statsleelab.github.io/gauss/articles/ref_33KG.html>).

**Supplementary Text 2.** The 33KG Reference Panel

GAUSS uses the 32,953 Genomes (33KG) reference panel ^1^. This panel contains 22,691 subjects from the Haplotype Reference Consortium (HRC) ^2^ and 10,262 subjects from CONVERGE ^3^, collectively representing a wide spectrum of ethnic backgrounds: 20,281 Europeans (EUR), 10,800 East Asians (ASN), 522 South Asians (SAS), 817 Africans (AFR), and 533 Native Americans (AMR). Although the HRC is predominantly European, it also incorporates data from 26 diverse populations as part of Phase 3 of the 1000 Genomes Project. Furthermore, an extra EUR population (ORK) was sourced from Orkney Island residents, who are also included in the HRC. In the case of CONVERGE, subjects were grouped into four populations based on their province of origin—China North East (CNE), China Central East (CCE), China South East (CSE), and China Central South (CCS)—all under the broader ASN super-population. A quadratic discriminant analysis model was employed to refine population labels, based on original labels and the first 20 ancestry principal components, potentially reassigning subjects to closely related populations^1^. Overall, the 33KG panel contains 29 populations under five super-populations: AFR, AMR, ASN, EUR, and SAS. Detailed subject counts, abbreviations, and population descriptions are provided in Supplementary Table 1.

**Supplementary Text 3.** Comparison of LD calculated using GAUSS’s computeLD() against LD computed from simulated individual-level data

To evaluate the performance and accuracy of the computeLD() function of the GAUSS software package, we conducted a series of simulation studies. These studies focused on three distinct scenarios of complex ancestry compositions, each involving a different 1Mbp genomic region:

**Scenario 1.** Composition of 50% ASW (African Ancestry in Southwest US) + 50% MXL (Mexican Ancestry from Los Angeles, USA) in the genomic region of chromosome 6: 50-51Mb.

**Scenario 2.** Composition of 60% CEU (Utah residents with Northern and Western European ancestry) + 20% ASW + 20% CCE (China Central East) in the genomic region of chromosome 11: 5-6Mb.

**Scenario 3.** Composition of 80% CCS (China Central South) + 10% ASW + 10% MXL in the genomic region of chromosome 19: 44.5-45.5Mb.

For each scenario, we simulated genotypes for 10,000 subjects reflective of the specified ancestry compositions. This involved drawing individual genotype data from the respective regions of the 33KG reference panel. For instance, in Scenario 1, we randomly sampled with replacement 5,000 ASW and 5,000 MXL subjects from the 33KG reference panel's ASW and MXL groups, respectively, within the Chr6: 50-51Mb region. Subsequently, LD was computed using this simulated individual-level genotype data. Then, we employed the computeLD() function for LD estimation in each scenario. The function was provided with inputs including the Illumina 1M SNP list of the 2022 PGC SCZ data, the ancestry composition, genomic location information, and the 33KG reference panel data.

The LD matrices obtained from the simulated individual-level genotype data were compared with those calculated by computeLD(). As illustrated in Supplementary Figure 1, the matrices exhibited a high degree of similarity, attesting to the accuracy of the computeLD() function. To quantitatively assess the correlation between the matrices in each scenario, we applied Mantel's test^4^. The results were consistently and strongly significant across all scenarios (simulated p-value = 0.0001 based on 9999 replicates), affirming the reliability of the computeLD() function in diverse genomic contexts.

**Supplementary Table 1.** The population group, the number of subjects, corresponding super population, and description for all 29 population groups in the 33KG reference panel. AFR: African, AMR: Admixed American, ASN: East Asian, EUR: European, SAS: South Asian

| **Population** | **Sample Size** | **Super Population** | **Description** |
| --- | --- | --- | --- |
| ACB | 164 | AFR | African Caribbeans in Barbados |
| ASW | 162 | AFR | African Ancestry in Southwest US |
| BEB | 86 | SAS | Bengali from Bangladesh |
| CCE | 3409 | ASN | China Central East |
| CCS | 2613 | ASN | China Central South |
| CDX | 95 | ASN | Chinese Dai in Xishuangbanna, China |
| CEU | 6360 | EUR | Utah residents with Northern and Western European ancestry |
| CLM | 98 | AMR | Colombians from Medellin, Colombia |
| CNE | 2330 | ASN | China North East |
| CSE | 2020 | ASN | China South-East |
| ESN | 140 | AFR | Esan in Nigeria |
| FIN | 3529 | EUR | Finnish in Finland |
| GBR | 2020 | EUR | British in England and Scotland |
| GIH | 110 | SAS | Gujarati Indian from Houston, Texas |
| GWD | 113 | AFR | Gambian in Western Divisions in the Gambia |
| IBS | 1309 | EUR | Iberian Population in Spain |
| ITU | 95 | SAS | Indian Telugu from the UK |
| JPT | 107 | ASN | Japanese in Tokyo, Japan |
| KHV | 226 | ASN | Kinh in Ho Chi Minh City, Vietnam |
| LWK | 99 | AFR | Luhya in Webuye, Kenya |
| MSL | 87 | AFR | Mende in Sierra Leone |
| MXL | 187 | AMR | Mexican Ancestry from Los Angeles, USA |
| ORK | 5772 | EUR | Orkney Island study |
| PEL | 110 | AMR | Peruvians from Lima, Peru |
| PJL | 121 | SAS | Punjabi from Lahore, Pakistan |
| PUR | 138 | AMR | Puerto Rican in Puerto Rico |
| STU | 110 | SAS | Sri Lankan Tamil from the UK |
| TSI | 1291 | EUR | Toscani in Italia |
| YRI | 52 | AFR | Yoruba in Ibadan, Nigeria |

**Supplementary Figure 1.** Comparison of LD matrices derived from simulated individual-level data versus GAUSS’s computeLD() function across varied ancestral compositions. The top row displays LD matrices estimated from simulated genotype data, while the bottom row shows LD matrices calculated using the computeLD() function from the GAUSS package. The comparison spans three different scenarios reflecting different ancestral contributions and genomic regions: Scenario 1: 50% ASW + 50% MXL in chr6: 50-51Mb; Scenario 2: 60% CEU + 20% ASW + 20% CCE in chr11: 5-6Mb; Scenario 3: 80% CCS + 10% ASW + 10% MXL in chr19: 44.5-45.5Mb. ASW: African Ancestry in Southwest US, CCE: China Central East, CCS: China Central South, CEU: Utah residents with Northern and Western European ancestry, MXL: Mexican Ancestry from Los Angeles, USA


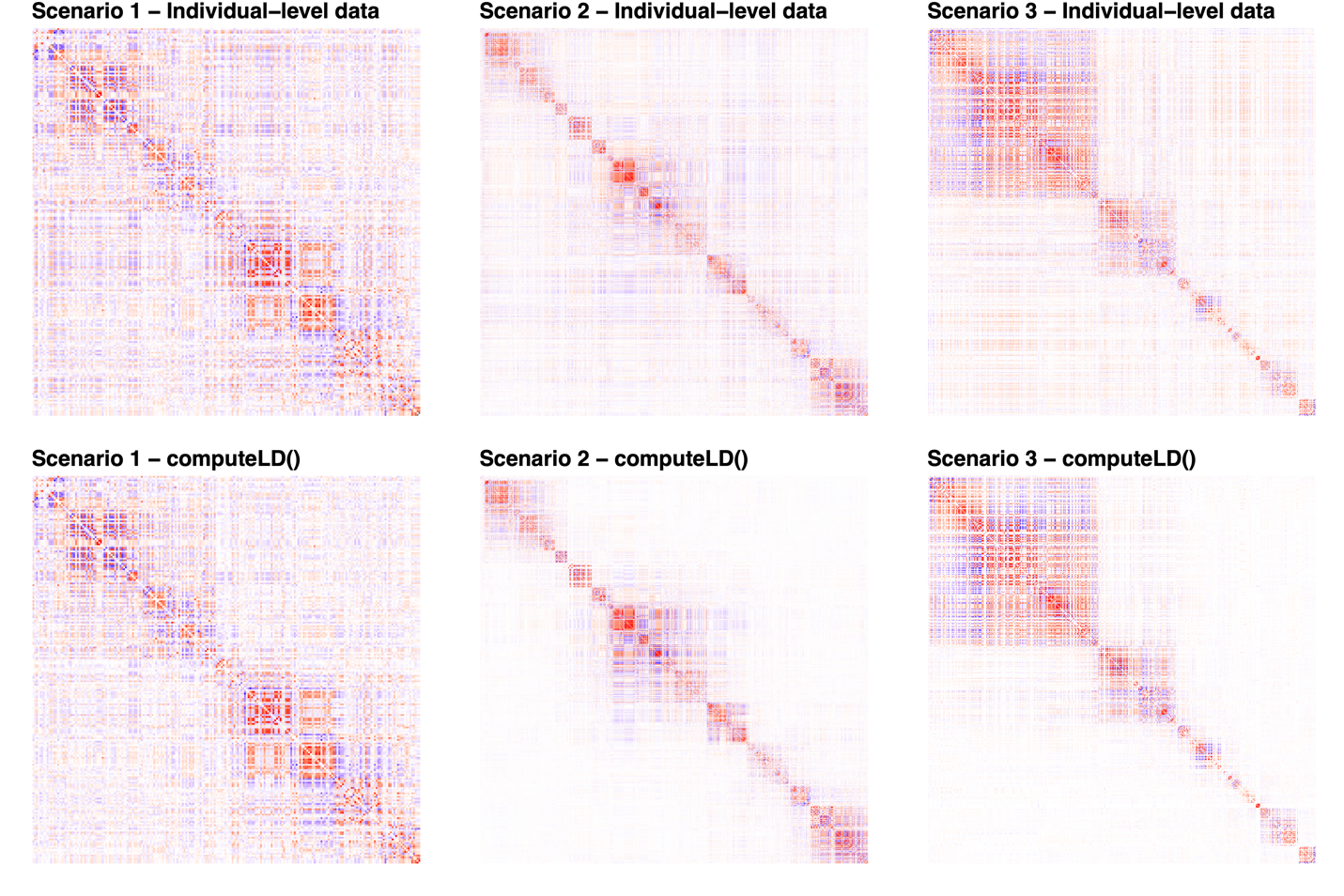


**Supplementary Figure 2.** Manhattan plot for Illumina 1M variants from the 2022 PGC SCZ summary data. This figure presents a Manhattan plot constructed using Illumina 1M variants extracted from the 2022 PGC SCZ summary data. The dataset, encompassing these specific variants, served as the basis for re-imputing the 2022 PGC SCZ study, utilizing the 33KG panel as detailed in Section 2.5.

**
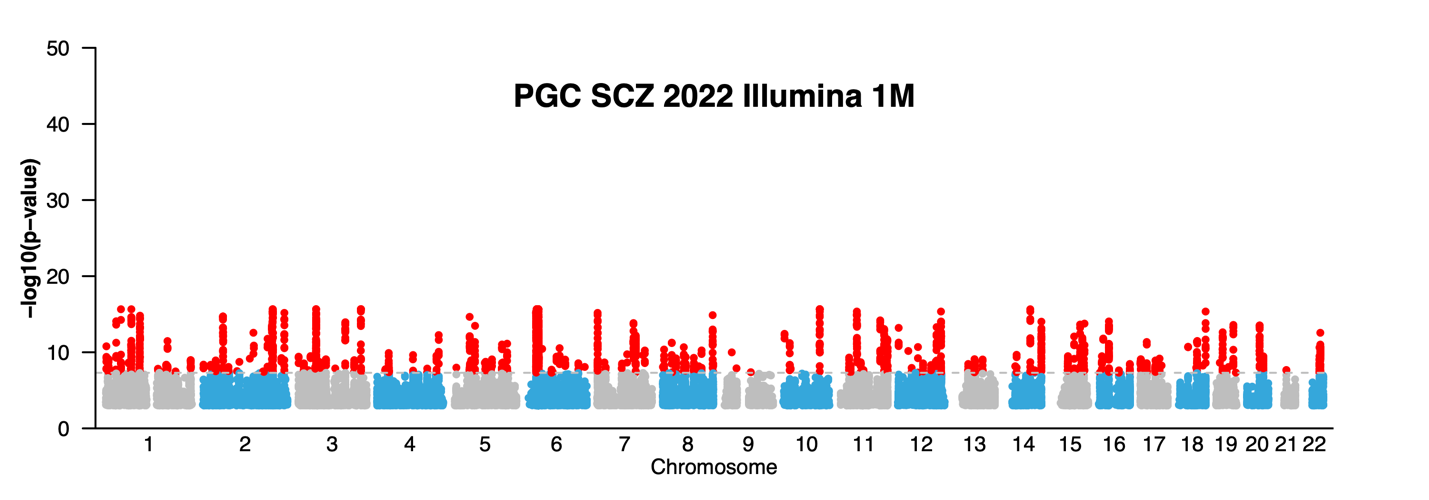
**

**References**

1. Chatzinakos, C. *et al.* Increasing the resolution and precision of psychiatric genome-wide association studies by re-imputing summary statistics using a large, diverse reference panel. *Am J Med Genet B Neuropsychiatr Genet* **186**, 16-27 (2021).

2. McCarthy, S. *et al.* A reference panel of 64,976 haplotypes for genotype imputation. *Nat Genet* **48**, 1279-83 (2016).

3. consortium, C. Sparse whole-genome sequencing identifies two loci for major depressive disorder. *Nature* **523**, 588-91 (2015).

4. Mantel, N. The detection of disease clustering and a generalized regression approach. *Cancer Res* **27**, 209-20 (1967).
